# Five selfish reasons to work reproducibly

**DOI:** 10.1186/s13059-015-0850-7

**Published:** 2015-12-08

**Authors:** Florian Markowetz

**Affiliations:** University of Cambridge, Cancer Research UK Cambridge Institute, Robinson Way, Cambridge, CB2 0RE UK

**Keywords:** Reproducibility, Scientific career

## Abstract

And so, my fellow scientists: ask not what you can do for reproducibility; ask what reproducibility can do for you! Here, I present five reasons why working reproducibly pays off in the long run and is in the self-interest of every ambitious, career-oriented scientist.

A complex equation on the left half of a black board, an even more complex equation on the right half. A short sentence links the two equations: “Here a miracle occurs”. Two mathematicians in deep thought. “I think you should be more explicit in this step”, says one to the other.

This is exactly how it seems when you try to figure out how authors got from a large and complex data set to a dense paper with lots of busy figures. Without access to the data and the analysis code, a miracle occurred. And there should be no miracles in science.

Working transparently and reproducibly has a lot to do with empathy: put yourself into the shoes of one of your collaboration partners and ask yourself, would that person be able to access my data and make sense of my analyses. Learning the tools of the trade (Box 1) will require commitment and a massive investment of your time and energy. A priori it is not clear why the benefits of working reproducibly outweigh its costs.

Here are some reasons: because reproducibility is the right thing to do! Because it is the foundation of science! Because the world would be a better place if everyone worked transparently and reproducibly! You know how that reasoning sounds to me? Just like yaddah, yaddah, yaddah …

It’s not that I think these reasons are wrong. It’s just that I am not much of an idealist; I don’t care how science should be. I am a realist; I try to do my best given how science actually is. And, whether you like it or not, science is all about more publications, more impact factor, more money and more career. More, more, more… so how does working reproducibly help me achieve more as a scientist.

## Reproducibility: what’s in it for me?

In this article, I present five reasons why working reproducibly pays off in the long run and is in the self-interest of every ambitious, career-oriented scientist.

### Reason number 1: reproducibility helps to avoid disaster

“How bright promise in cancer testing fell apart” titled a *The New York Times* article published in summer 2011 [1] highlighting the work of Keith Baggerly and Kevin Coombes, two biostatisticians at M.D. Anderson Cancer Center. Baggerly and Coombes had exposed lethal data analysis problems in a series of high-impact papers by breast cancer researchers from Duke University [2].

The issues discovered by Baggerly and Coombes could have easily been spotted by any co-author before submitting the paper. The data sets are not huge and can easily be spot-checked on a standard laptop. You do not have to be a statistics wizard to realize that patient numbers differ, labels got swapped or samples appear multiple times with conflicting annotations in the same data set. Why did no one notice these issues before it was too late? Because the data and analysis were not transparent and required forensic bioinformatics to untangle [2].

For me, this example provides a powerful motivation to be more transparent and reproducible in my own work. Even smaller disasters can be embarrassing. Here is an example from my own research. Our experimental collaboration partners were validating a pathway model that we had generated computationally. When writing the paper, however, we hit a crucial roadblock: no matter how hard we tried, we could not reproduce our initial pathway model. Maybe the data had changed, maybe the code was different, or maybe we just couldn’t remember the parameter settings of our method correctly. Had we published this result, we would not have been able to demonstrate how the validated hypothesis was generated from the initial data. We would have published a miracle.

This experience showed me two things. First of all, a project is more than a beautiful result. You need to record in detail how you got there. And second, starting to work reproducibly early on will save you time later. We wasted years of our and our collaborators’ time by not being able to reproduce our own results. All of this could have been avoided by keeping better track of how the data and analyses evolved over time.

### Reason number 2: reproducibility makes it easier to write papers

Transparency in your analysis makes writing papers much easier. For example, in a dynamic document (Box 1) all results automatically update when the data are changed. You can be confident your numbers, figures and tables are up-to-date. Additionally, transparent analyses are more engaging, more eyes can look over them and it is much easier to spot mistakes.

Here is another example from my own work. In a different project [3], a collaborating clinician and I were discussing why some survival results in a multi-centre study did not come out as expected. Because all the data and analysis code were available to us in an easy-to-read file, we could explore the question ourselves. By simply generating a table of the variable describing tumor stage, we were able to spot the problem: what we expected to see were the stage numbers 1–4, what we saw were entries like ‘XXX’, ‘Fred’ and ‘999’. The people who had given us the data had apparently done a poor job in curating it. Looking into the data ourselves was much quicker and more engaging than going to the postdoc working on the project and saying, ‘Figure this out for us’. My collaborator and I are much too busy to spend too much time on low-level data cleaning, and without the well documented analysis we would not have been able to contribute; but because we had very transparent data and code, it cost us just five minutes to spot a mistake.

### Reason number 3: reproducibility helps reviewers see it your way

Most of us like to moan about peer review. One of the complaints I hear most often is: the reviewers didn’t even read the paper and had no idea what we were really doing.

This starkly contrasts with my experience during the review process of a recent paper [4], for which we had made the data and well-documented code easily accessible to the reviewers. One of the reviewers proposed a slight change to some analyses, and because he had access to the complete analysis, he could directly try out his ideas on our data and see how the results changed. The reviewer was completely on board, the only thing left to discuss was the best way to analyze the data. Exactly how a constructive review should be. And it would have been impossible without a transparent and reproducible presentation of our analyses.

### Reason number 4: reproducibility enables continuity of your work

I would be surprised if you hadn’t heard the following remarks before, maybe you have even said them yourself: “I am so busy, I can’t remember all the details of all my projects” or “I did this analysis 6 months ago. Of course I can’t remember all the details after such a long time” or “My principle investigator (PI) said I should continue the project of a previous postdoc, but that postdoc is long gone and hasn’t saved any scripts or data”.

Think about it, all of these issues can be solved by documenting data and code well and by making them easily accessible. This point is particularly important for PIs who work on challenging long-term projects. How can you ensure the continuity of work in your lab if progress is not documented reproducibly? In my own group, I don’t even discuss results with students if they are not documented well. No proof of reproducibility, no result!

### Reason number 5: reproducibility helps to build your reputation

For several papers, we have made our data, code and analyses available as an Experiment Package on Bioconductor [5]. When I came up for tenure, I cited all of these packages as research output of my lab. Generally, making your analyses available in this way will help you to build a reputation for being an honest and careful researcher. Should there ever be a problem with one of your papers, you will be in a very good position to defend yourself and to show that you reported everything in good faith.

The recent paper published in *Science* “Scientific standards. Promoting an open research culture” [6] summarizes eight standards and three levels of reproducibility guidelines. Using tools such as R and knitR (Box 1) will make it likely that you comply easily with the highest-level guideline — and again, that is good for your reputation.

## What’s holding you back?

Have I convinced you? Maybe not. Here is a collection of responses I sometimes get to my insistence on reproducible research (as well as my answers to them):

“*It’s only the result that matters*!” You are wrong.

“*I’d rather do real science than tidy up my data*”. If you don’t work reproducibly, you are not doing science at all [7].

“*Mind your own business! I document my data the way I want!*” Yes, please do! There are many ways to work reproducibly [8] and you can pick whatever suits you best.

“*Excel works just fine. I don’t need any fancy R or Python or whatever*”*.* The tool you mention might work well if lots of manual curation is needed, but as soon as you do data analysis, less clicking and more scripting are the way to go. Imagine you have to do a simple analysis such as a regression plot 5 times (10 times, 20 times) and compare doing it by hand 5 times (10 times, 20 times) to writing a simple loop to do it for you. Now imagine having to do it again 3 weeks later because the data have slightly changed. R and Python are clearly the way to go.

“*Reproducibility sounds alright, but my code and data are spread over so many hard drives and directories that it would just be too much work to collect them all in one place*”*.* Just think about what you just said. Your lack of organization puts you and your project in grave danger.

“*We can always sort out the code and data after submission*”*.* My pathway example above shows the danger of this strategy. Also, preparing a manuscript for submission can take a long time and you might not even remember all the details of your analysis by the time you submit your results.

“*My field is very competitive and I can’t risk wasting time*”*.* And that is exactly why you should start working reproducibly early on, so you don’t waste time in the long run.

## When do you need to worry about reproducibility?

Let’s assume that I have convinced you that reproducibility and transparency are in your own best interest. Then what is the best time to worry about it?

Long answer: before you start the project, because you might have to learn tools like R or git. While you do the analysis, because if you wait too long you might lose a lot of time trying to remember what you did two months ago. When you write the paper, because you want your numbers, tables and figures to be up to date. When you co-author a paper, because you want to make sure that the analyses presented in a paper with your name on are sound. When you review a paper, because you can’t judge the results if you don’t know how the authors got there.

Short answer: Always!

## Achieving a culture of reproducibility

Who are reproducibility and transparency important for? Obviously, students and postdocs play a major part in reproducible work, because more often than not they are the people who actually do the work. My advice is: learn the tools of reproducibility (Box 1) as quickly as possible and use them in every project. You will get many benefits out of these efforts: you will make fewer mistakes and more easily correct those that you do make; you will be more efficient and in the long run progress much faster; and if you think your supervisor is too hands-off, making your analyses more accessible is a good strategy to help them be more engaged.

PIs, group leaders, professors, team leaders — it is up to you to build a ‘culture of reproducibility’ on top of the technical foundations your students and postdocs have laid. In my own lab, I have made reproducibility a key point in a document that I hand out to new starters [9]. A simple strategy to show your support is to ask for documentation of analysis every time a team member shows you their result. You don’t have to go into the details; a quick look will tell you how well it is done. What has really improved reproducibility in my own lab is to require that, before paper submission, a team member not involved in the project tries to independently run the analyses and reproduce our results.

If you fail to create a culture of reproducibility in your lab, you will miss out on the large scientific pay-offs that reproducibility offers in the long run.

Science is becoming more transparent and reproducible every single day. You can be a leader in this process! A cutting-edge trend-setter! Come on, I know you want it too.

## Twitter and blog

Follow Florian on Twitter @markowetzlab and on his blog http://scientificbsides.wordpress.com

## Box 1

At the lowest level, working reproducibly just means avoiding beginners’ mistakes. Keep your project organized, name your files and directories in some informative way, store your data and code at a single backed-up location. Don’t spread your data over different servers, laptops and hard drives.

To achieve the next levels of reproducibility, you need to learn some tools of computational reproducibility [8]. In general, reproducibility is improved when there is less clicking and pasting and more scripting and coding. For example, do your analysis in R (https://www.r-project.org/) or Python (https://www.python.org/) and document your analysis using knitR (http://yihui.name/knitr/) or IPython notebooks (http://ipython.org/). These tools help you to merge descriptive text with analysis code into dynamic documents that can be automatically updated every time the data or code change.

As a next step, learn how to use a version-control system like git (https://git-scm.com/) on a collaborative platform such as GitHub (https://github.com/). Finally, if you want to become a pro, learn to use docker (http://www.docker.com/), which will make your analysis self-contained and easily transportable to different systems.
